# Collagenous Gastritis: A Cause of Pediatric Iron Deficiency Anemia

**DOI:** 10.14309/crj.0000000000001000

**Published:** 2023-03-04

**Authors:** Conner Blackmore, Margaret Leach

**Affiliations:** 1 South Western Sydney Clinical School, University of New South Wales, Sydney, Australia; 2 School of Medicine Notre Dame, Fremantle, Australia

**Keywords:** iron deficiency anemia, stomach, auto-immune

## Abstract

Collagenous gastritis (CG) is a rare histopathological condition characterized by subepithelial collagen deposition and inflammatory infiltrates in the gastric mucosa. With less than 100 cases reported in current literature, clinical presentation is highly variable. We report a case of isolated CG in an 11-year-old girl who presented with a 6-month history of symptomatic severe iron deficiency anemia (nonexertional shortness of breath, palpitations, chest pain, and lethargy). CG is a rare condition in children require long-term follow-up and monitoring of their disease; and because of its rarity, a targeted treatment does not exist. The current therapeutic strategy is focused on symptom control and monitoring iron studies, in conjunction with regular follow-up.

## INTRODUCTION

Collagenous gastritis (CG) is a rare histopathological condition characterized by subepithelial collagen deposition and inflammatory infiltrates within the lamina propria of the gastric mucosa.^1,2^ In the current literature, less than 300 cases have been reported, with the first case reported in 1989.^3–5^ Currently, 2 phenotypes of the disease have been described: pediatric-onset and adult-onset types, with the adult-onset type associated with collagenous colitis and autoimmune disorders.^6^ Diagnosis is based on histological evidence of increased (>10 μm) subepithelial collagen deposition in addition to endoscopic findings (mucosal erythema, nodularity, and ulceration) and a high clinical suspicion.^1,5^ The most common clinical presentation of pediatric CG is severe symptomatic iron deficiency anemia (fatigue, pallor, palpitations, and dyspnea), with less common presentations including weight loss, abdominal pain, dyspepsia, and gastrointestinal bleeding.^1,6^

## CASE REPORT

An 11-year-old girl with a long history of iron deficiency anemia was referred to the hospital for medical review with a 6-week history of worsening nonexertional shortness of breath, palpitations, chest pain, and reduced exercise tolerance. Her dyspnoea was initially believed to be contributed to episodes of hyperventilation with anxiety and were not associated with exercise. Usually, she exercised 7 times per week; however, on admission, a significant reduction in her usual exercise tolerance with profound lethargy after just 2 sessions was reported. Her palpations were worsening over the 6 weeks before admission, which were described as intermitted, self-resolving, and not associated with exercise. Similarly, during this period, she reported chest pain, describing it as “sharp, burning, or heavy.” This was self-resolving and not associated with either palpitations or exercise. From the patient's and her parent’s collateral history, there was no suggestive cause of anemia. She was also premenarchal and eating a varied, nonrestrictive diet, which included red meat 5 times per week. It was notable that her father and maternal grandmother had autoimmune conditions of alopecia and Hashimoto thyroiditis. Previously, the adult phenotype of CG has predominantly been associated with autoimmune disorders. This hospital admission, she received a transfusion of packed red blood cells, which improved her dyspnoea, lethargy and palpations; was commenced on oral iron and vitamin C supplementation and outpatient follow-up with a general paediatrician was arranged.

**Figure 1.**
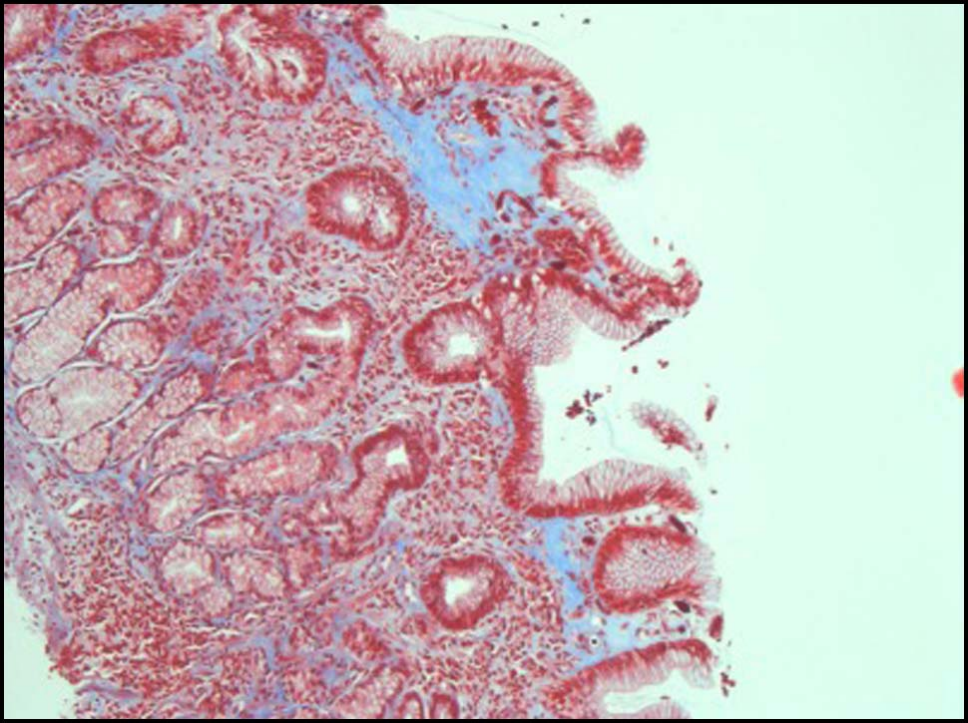
Gastric biopsy with Masson trichrome stain (200× magnification) demonstrating thickened collagen bands in blue.

**Figure 2.**
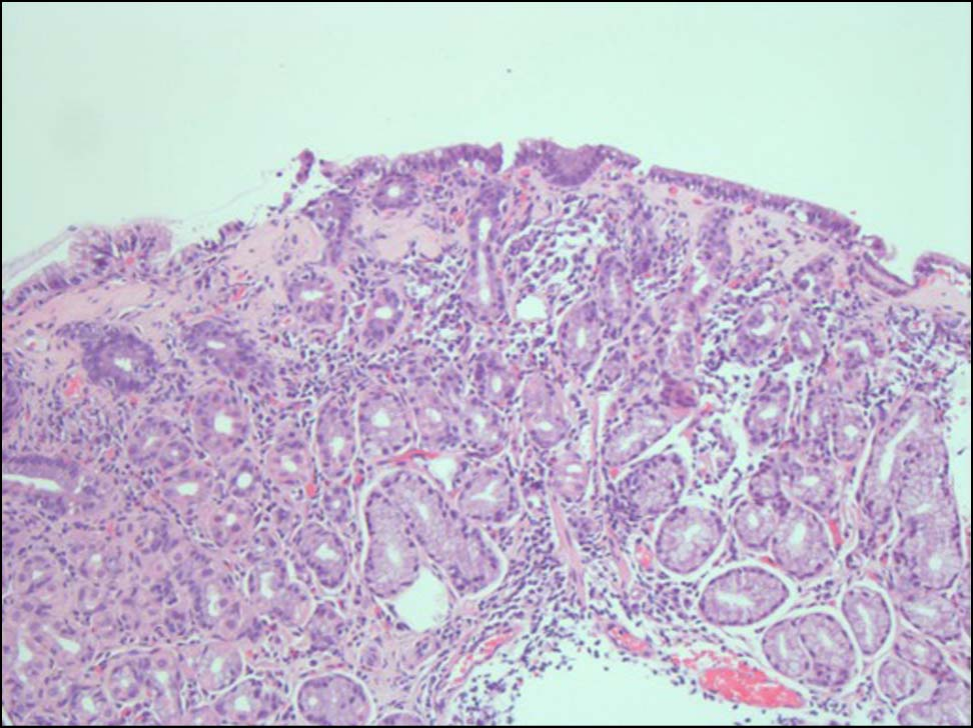
Duodenal biopsy with hematoxylin and eosin stain (200× magnification), demonstrating villous epithelial reactive changes with patchy, moderate intraepithelial lymphocytosis.

Recurrent persistent symptoms led to further workup, including celiac serology; thyroid function tests; and total immunoglobulin (Ig) A, IgM, and IgG, all of which returned normal. The patient was referred to a tertiary center at 14 years for further evaluation by a gastroenterologist. An esophagogastroduodenoscopy identified 4 regions of superficial gastritis measuring from 5 to 7 mm. Five random esophageal, gastric, and duodenal biopsies were obtained. Esophageal biopsies demonstrated squamous mucosa with no evidence of inflammation, hyperplasia, or fungal colonization, using periodic acid-Schiff stain. Gastric biopsies confirmed no active gastritis or *Helicobacter pylori* infection, using Loeffler methylene blue stain. There was evidence of patchy but focally increased hyaline collagen deposition (confirmed with Masson trichrome stain) within the superficial lamina propria, extending to just beneath the superficial epithelium associated with trapped lymphocytes (Figure 1). There was no evidence of duodenitis on duodenal biopsies; however, villous surface epithelium indicated reactive changes with patchy, overall-moderate intraepithelial lymphocytosis extending to the tips of the villi with no identified organism (Figure 2). These histopathological findings in association with the patient's clinical history are most consistent with a diagnosis of CG.

## DISCUSSION

CG is a rare diagnosis in children, with diverse clinical presentation (Table 1) and unknown pathophysiology. Current literature suggests that the most common presentation of pediatric CG is severe symptomatic iron deficiency anemia with a higher predominance in the female population, as was seen in this case.^2,6,7^ The etiology of CG is currently unknown. There are currently 3 main hypotheses for the underlying pathogenic mechanism of CG, which include (i) chronic inflammation, (ii) fibroblast sheath abnormalities, and (iii) leakage of plasma proteins and fibrinogen.^1,2^ Other intestinal and autoimmune disorders, including celiac disease, collagenous enteritis, collagenous colitis, Sjogren syndrome, systemic lupus erythematosus, juvenile arthritis, rheumatoid arthritis, Hashimoto thyroiditis, Graves' disease, diabetes mellitus type 1, and common variable immune deficiency, have been predominantly associated with the adult phenotype of CG; however, increasing evidence suggests that this link also exists with the pediatric phenotype.^2,8–10^ This suggests that CG may share a common autoimmune mechanism in pathogenesis with these diseases.

**Table 1.** Clinical presentations of collagenous gastritis^4,7^

Most common
Anemia—symptomatic (fatigue/lethargy, pallor, palpitations, and dyspnea) and incidental
Abdominal pain
Dyspepsia
Less common
Gastrointestinal bleeding
Nausea and vomiting
Vertigo
Nonbloody diarrhea
Weight loss

The current gold standard for the diagnosis of CG is upper endoscopy with gastric biopsy with endoscopic appearance, which include nodularity, mucosal erythema, hemorrhage, or ulceration.^2,3^ Subepithelial thickening of the collagen band, at least 10 μm in thickness, is a characteristic histological finding of CG.^2,11^ According to current literature, there are 3 described patterns of inflammation—lymphocytic gastritis-like pattern, eosinophilic-rich pattern, and atrophic pattern.^10^ Although endoscopy is a widely used technique, miss rates are believed to be high because of the subtle histological patterns associated with CG.^2^

There are currently no established treatment guidelines for CG, with numerous medications trialed with variable responses. Trialed treatments include dietary modifications (eg, gluten-free diet), corticosteroids, H2-receptor blockers, misoprostol, proton-pump inhibitors, sucralfate, oral iron replacement, and 5-aminosalycylates.^2,12^ Current key focus for the management of CG in the literature is identification and treatment of symptoms to improve the patient’s overall quality of life.^4,7^ In this case, there was a focus on monitoring and maintaining appropriate iron stores, to address the severe symptomatic iron deficiency anemia, and regular long-term follow-up, because of the high suspicion for additional autoimmune diseases. The natural history and long-term progression of CG is variable, regardless of the pediatric or adult phenotype. In some cases of CG, there is complete resolution; however, the majority seems to have a chronic persistent disease pattern.^9^ According to some literature, it has been suggested that disease course and severity may be associated with collagen thickness.^12^ Currently, there is no definitive information on the long-term progression of CG or treatment regimen, hence the need for continuous follow-up and monitoring of the disease.

In summary, we present another case of CG to add to the limited literature of cases of this rare condition. The symptoms in our patient had been consistent with current literature; however, there was a strong family history of autoimmune conditions, not commonly associated with the pediatric phenotype of CG. We highlighted symptom control as the key focus of current treatment of CG in the absence of a targeted therapy, to improve the patient's overall quality of life, with regular long-term follow-up and monitoring of the disease. We hope to raise awareness among clinicians to consider CG in the differential diagnosis for a young female patient presenting with symptomatic anemia and gastrointestinal symptoms. Our case highlights the need for increased literature on rare diseases such as CG to improve awareness, to improve accuracy of disease information, and to develop treatment and monitoring guidelines.

## DISCLOSURES

Author contributions: C. Blackmore: writing, editing, final approval, and submission. M. Leach: writing and editing.

Financial disclosure: None to report.

Informed consent was obtained for this case report.
